# Validation of Scoring Tool for the Lipid Profile Interpretation in Exercise Training: SLIEX

**DOI:** 10.21315/mjms-11-2024-889

**Published:** 2025-04-30

**Authors:** Hashbullah Ismail, Wahidah Tumijan, Mazlifah Omar, Sazzli Shahlan Kasim, Shamshuritawati Sharif, Noor Fatihah Ilias

**Affiliations:** 1 Faculty of Sports Science and Recreation, Universiti Teknologi MARA, Shah Alam, Selangor, Malaysia; 2 Faculty of Sports Science and Recreation, Universiti Teknologi MARA, Seremban, Negeri Sembilan, Malaysia; 3 Faculty of Medicine, Universiti Teknologi MARA, Sungai Buloh, Selangor, Malaysia; 4 Cardiovascular Advancement and Research Excellence Institute (CARE Institute), Universiti Teknologi MARA, Sungai Buloh, Selangor, Malaysia; 5 Collage of Arts and Sciences, Universiti Utara Malaysia, Sintok, Kedah, Malaysia

**Keywords:** scoring tool, exercise intervention, lipid profiles, health professionals, reliability

## Abstract

**Background:**

Interpreting the effects of exercise is complicated because lipid profiles contain four different markers. Thus, scoring tools are required to aid interpretation of the effects of exercise on lipid profiles. A Scoring tool for Lipid profile Interpretation in EXercise (SLIEX) was designed specifically for use by health professionals to interpret effects of exercise intervention on lipid profiles.

**Methods:**

The tool consisted of 18 scores (4 scores for changes pre- and post-intervention, and 14 scores for weightage of changes). This score provides the proportion of improvement in the lipid profile following exercise intervention. Kappa statistics (κ) were used to measure interobserver agreement, and the interclass correlation coefficient (ICC) was used to check the reliability of the scoring tool. One-way ANOVA was used to identify systematic differences between observers using the Statistical Package for the Social Sciences (SPSS) version 28 with statistical significance set at *P* < 0.05.

**Results:**

The summated SLIEX scores for each observer showed no systematic differences [F (2, 69) = 0.09, *P* = 0.991]. The summated SLIEX scores of the three observers showed significant association and excellent agreement as follows: Observers 1 and 2: ICC = 0.950, 95% confidence interval (CI), 0.889–0.978, *P* < 0.001; Observers 2 and 3: ICC = 0.993, 95% CI, 0.983–0.997, *P* = 0.000; Observers 1 and 3: ICC = 0.972, 95% CI, 0.937–0.988, *P* < 0.01.

**Conclusion:**

The SLIEX score is a new and reliable tool designed for health professionals to interpret the effect of exercise intervention on lipid profiles.

## Introduction

Despite advancements in the last several decades in the understanding and treatment of cardiovascular disease, atherosclerotic cardiovascular disease (ASCVD) remains the leading cause of morbidity and mortality (1). The ASCVD is the primary cause of coronary artery disease (CAD) (2). The gradual build-up of atherosclerotic plaques within an epicardial coronary artery causes attenuation of myocardial perfusion, known as CAD (3). Dyslipidaemia, defined as an abnormal blood lipid profile, is the main risk factor for CAD. Increased plasma levels of triglycerides (TG), total cholesterol (TC), and low-density lipoprotein cholesterol (LDL-C) levels and decreased plasma levels of high-density lipoprotein cholesterol (HDL-C) are the hallmarks of dyslipidaemia (4). Measuring the serum levels of fasting TG, TC, LDL-C, and HDL-C is part of the conventional method for determining dyslipidaemia risk factors for CAD (5, 6). Diagnosing dyslipidaemia involves taking a serum lipid profile, which describes the different amounts of lipids in the blood.

Exercise is an established therapeutic strategy in management and prevention. However, much remains to be learned regarding the optimal exercise regimen for lipid profile management. There are inconsistent findings regarding the effects of exercise on lipid profiles. An improvement in the lipid profile post-exercise has been noted in some studies; yet, other studies have shown that exercise has no positive effect. However, the effects of exercise on blood lipid levels remain controversial (7). One factor contributing to these inconsistent findings is the diversity of exercise training prescribed and different lipid profiles (8). As shown in Table 1, involving 12 randomised controlled trials [RCTs] (9–20) among patients with CAD undergoing a variety of aerobic exercises, including various weekly frequencies, different types, durations, and exercise intensities, were designed. The results revealed that exercise has varying effects on lipid profiles.

Designed exercise training commonly uses the FITT principles, which represent frequency (F), intensity (I), time (T), and type (T). Among these four principles, intensity plays a major role in determining exercise effectiveness (21, 22). As seen in Table 1, the data show that the effect of different intensities, namely high intensity interval training (HIIT) and moderate intensity continuous training (MICT) on lipid profiles among patients with CAD was genuinely inconsistent. These 12 studies were randomly selected from a list of RCTs comparing two different interventions, HIIT and MICT, in certain periods to compare which intervention is most effective among patients with CAD.

Moreover, dyslipidaemia was characterised by four different markers, and the results were expected to decrease TG, TC, and LDL-C levels and increase HDL-C levels to indicate effectiveness. However, interpreting the effects of exercise became complicated because some markers improved, whereas others worsened. Owing to these conflicting results, it is difficult to decide which intensity, HIIT or MICT, is most beneficial for controlling lipid profiles among patients with CAD. As shown in Table 1, a study (10) involving a large sample size showed that both HIIT and MICT improved TG and HDL-C levels and worsened TC and LDL-C levels. Next, another study (20) found that HIIT improved the TG, LDL-C, and HDL-C levels, but did not change TC levels, while MICT improved LDL-C and HDL-C levels and worsened TG and TC levels. Another research (16) showed that HIIT improved TG, LDL-C, and HDL-C levels and worsened TC levels, whereas MICT improved HDL-C levels and worsened other markers. These conflicting results were observed in all 12 RCTs involving patients with CAD. This raises several questions. The first is whether exercise is still deemed helpful if it only improves two markers, while negatively affecting two other markers. Second, a query arises as to how these four distinct lipid profile indicators should be interpreted to determine exercise benefits. Finally, the intensity at which the lipid profile improves the most remains to be checked.

Thus, there is no scoring tool available as of yet that can be used to quantify the benefits of exercise on lipid profiles. Therefore, this study’s primary objective was to develop an exercise science lipid profile scoring tool designed for health professionals to interpret the effectiveness of exercise using four different lipid profile markers. The secondary objective was to assess the validity and reliability of the scoring tools. The scoring tool was designed to assess the effects of exercise programmes for patients with CAD and other health problems. By developing this Scoring tool for Lipid profile Interpretation in EXercise (SLIEX), this study aimed to improve optimal exercise training design for managing dyslipidaemia risk factors and indirectly improve lipid profile markers.

## Methods

The authorship group consisted of members of a research team with experience in conducting exercise training for the chronic disease group and examining lipid profile markers. The authors comprised clinical exercise physiologists, rehabilitation medicine specialists, cardiac rehabilitation specialists, and cardiologists. The consensus among all members was that a new tool needs to be developed to interpret the impact of exercise on lipid profiles due to widely varying results. A series of meetings was organised to discuss the scoring tool content. The content was developed based on agreement of all team members. After content finalisation, a draft protocol was prepared, and its reliability was assessed.

### SLIEX Score Criteria

The overall benefit of exercise for all dyslipidaemia factors was calculated by scoring changes and the weightage of changes for all lipid profile markers. The first component was any change in the pre- and post-intervention results. If there is an improvement (decrease in TG, TC, and LDL-C and increase in HDL-C), 1 mark is given, 0 marks for no changes pre- and post-test, and −1 mark for a worsening effect for all markers. The total score was −4 to 4 for all markers found between the pre- and post-tests.

The second component was measuring the weight of changes. Changes in weightage assessed the efficacy of the intervention by scrutinising the enhancement of the category from pre-test to post-test. The weightage of changes was classified based on the Third Report of the National Cholesterol Education Program (NCEP) Expert Panel on Detection, Evaluation, and Treatment of High Blood Cholesterol in Adults (Adult Treatment Panel III) (23). Table 2 shows the norm for dyslipidaemia markers. A mark was assigned based on the weight changes by category (normal, near optimal, borderline high, high, and very high) for all markers. The total weightage scores were: TG, −3 to 4; LDL-C, −4 to 5; HDL-C, −1 to 2, and TC, −2 to 3.

Table 3 refers to the range of scores for TG. Notably, one score is given for no changes in category from baseline to post-test. One score was considered the minimal benefit of exercise, in which exercise did not cause worsening. Two scores were given for each improvement in categories from baseline to post-test. For example, if TG at baseline was borderline high, and the post-test improved to normal, two scores were given. Next, three scores were given for two improvements in the categories from baseline to post-test. For example, if the TG baseline was high, and the post-test improved to normal, three scores were assigned. Four scores were given for three category improvements from baseline to post-test. For example, if the TG baseline was very high, and the post-test improved to normal, four scores were given. Scores of −1 to −3 were assigned to the worsening effect after exercise. Worsening in one, two, and three categories from baseline to post-test were given scores of −1, −2, and −3, respectively. The higher the score, the better the results. The maximum score for TG was 4, and the lowest was −3.

Table 4 shows the range of scores for LDL-C. A score of one indicated no change in category from baseline to post-test. One score was considered the minimal benefit of exercise, in which exercise did not cause worsening. Two scores were given for each improvement in categories from baseline to post-test. For example, if the LDL-C baseline was near optimal, and the post-test improved to normal, two scores were given. Three scores were given for two improvements in the categories from baseline to post-test. For example, if the LDL-C baseline was borderline high, and post-test improved to normal, three scores were given. Meanwhile, four scores were given for three category improvements from baseline to post-test. For example, if the LDL-C baseline was high, the post-test improved to normal, four scores were given. Next, five scores were given for four category improvements from baseline to post-test. For example, if the LDL-C baseline was very high, and the post-test improved to normal, five scores were given. Notably, −1 to −4 marks indicate a worsening effect after exercise. In case of worsening in one, two, three, and four categories from baseline to post-test, scores of −1, −2, −3, and −4 were given, respectively. The higher the score, the better the results. The maximum score for LDL-C was 5, and the lowest was −4.

Table 5 shows the HDL-C range scores. A score of one indicated no change in category from baseline to post-test. One score was considered the minimal benefit of exercise, in which exercise did not cause worsening. Two scores were given for each improvement from the abnormal to normal HDL-C categories. On the other hand, a score of −1 was given for changes from normal to abnormal. For the abnormal category, a score of 0 was given if there were no changes from baseline to post-test. The higher the score, the better the results. The maximum score for HDL-C was 2, and the lowest was −1.

Table 6 shows the range of the TC scores. A score of one indicated no change in category from baseline to post-test. One score was considered the minimal benefit of exercise, in which exercise did not cause worsening. Two scores were given for each improvement in categories from baseline to post-test. For example, if the TC baseline was borderline high, and the post-test improved to normal, two scores were given. Meanwhile, three scores were given for two improvements in the categories from baseline to post-test. For example, if the TC baseline was high, and the post-test improved to normal, three scores were given. Scores of −1 to −2 were assigned for worsening effects after exercise. Worsening in one and two categories from baseline to post-test were given scores of −1 and −2, respectively. The higher the score, the better the results. The maximum scores for TG were 3 and the lowest was −2.

The total score for both components was 18, with 4 scores for improvement seen pre- and post-test, and 14 scores for weight changes. The total score obtained was divided by 18 and then multiplied by 100 to calculate the percentage of improvement. Using this score, improvements in the lipid profile after exercise can be measured by evaluating the percentage of improvement. The overall score for incomplete data depends on the total score of the available data. Therefore, to quantify the effect of exercise on lipid profile, a greater improvement in the lipid profile is required. The results can be interpreted into four categories: < 25% improvement is considered as fair improvement, 25%–50% as good improvement, > 50%–75% as very good improvement, and > 75% as excellent improvement.

### Reliability of SLIEX

Three observers (NFI, MO, and HI) independently evaluated the total SLIEX scores of 12 published articles comparing HIIT and MICT exercise training on lipid profiles among patients with CAD using the SLIEX score. All observers had experience conducting exercise training studies and varying levels of expertise in assessing the quality of exercise intervention trials. Studies were randomly selected to examine the effect of aerobic exercise between HIIT and MICT on lipid profiles in patients with CAD. All selected studies were RCTs. Overall, there were 18 available scores (4 scores for changes pre- and post-intervention and 14 scores for weightage of changes). However, if the data are incomplete under certain circumstances, the total score can still be determined using the available data. For example, if the data for TC were incomplete, they were excluded from the analysis, yielding a total score of 14. In the SLIEX tool, observers assign a score according to the guidelines for all parameters. Each observer was provided with a copy of the SLIEX score guidelines, 12 research papers, and a standardised Excel spreadsheet to record data. All observers will explain the guidelines for using this tool in detail.

#### Statistical Analysis of Reliability

The inter-observer agreement between each observer (*n* = 3) was assessed for each individual point available on the SLIEX scale to find an agreement of the total score for the SLIEX tool (pre-and post-change = 4 scores, and weighted changes = 14 scores, 18 scores in total) using the Cohen Kappa statistic (κ). Kappa statistics are appropriate for measuring the agreement between individuals when the data are nominal. The use of these statistics is consistent with a previous study (24) that assessed interobserver agreement in the tool for the assessment of study quality and reporting in exercise (TESTEX) score. The Kappa result can be interpreted as follows: values ≤ 0.00 indicating no agreement and 0.01–0.20 as none to slight, 0.21–0.40 as fair, 0.41–0.60 as moderate, 0.61–0.80 as substantial, and 0.81–1.00 as almost perfect agreement (25).

The reliability of the total score for each observer was assessed using intraclass correlation coefficients (ICCs) and the associated 95% confidence intervals (95% CIs). Values < 0.5 indicate poor reliability, values ≥ 0.5 to < 0.75 indicate moderate reliability, values between ≥ 0.75 to < 0.9 indicate good reliability, and values ≥ 0.9 indicate excellent reliability (26). Systematic differences between the three observers were evaluated using a parametric test with one-way ANOVA after checking the normality test. Data analyses were performed using the Statistical Package for the Social Sciences (SPSS) version 28 (IBM Corp., Armonk, NY, US), and statistical significance was set at *P* <0.05.

As presented in Table 7, the agreement (κ) between observers 1 and 2 ranged between 0.733 (substantial) and 1.00 (almost perfect). Substantial agreement was observed in the pre-and post-HDL-C changes and weight changes in the TG group. Near-perfect constant values of approximately 100% agreement occurred in six of the eight categories (approximately 75%). Observers 2 and 3 and observers 1 and 3 achieved almost perfect agreement in seven of the eight categories (~87.5%) and substantial agreement in only one. The results showed substantial to almost perfect agreement among all observers.

There was a significant association among the summated SLIEX scores of the three observers, with excellent agreement among all observers as follows: observers 1 and 2, ICC = 0.950, 95% CI, 0.889–0.978, *P* < 0.001; observers 2 and 3, ICC = 0.993, 95% CI, 0.983–0.997, *P* = 0.000; and observers 1 and 3, ICC = 0.972, 95% CI, 0.937–0.988, *P* < 0.01. The results showed excellent reliability of the scoring tool among all observers. Additionally, there were no systematic differences between the summated SLIEX scores for each observer F (2, 69) = 0.09, *P* = 0.991.

## Discussion

### Usability of SLIEX Scoring

This study has developed a new scoring tool designed for use by health professionals in clinical practice to assist in interpreting the effect of exercise intervention on lipid profiles. The SLIEX tool consists of 18 scores, and the results can be translated into four categories: fair, good, very good, and excellent.

The SLIEX scoring tool is advantageous in that it allows to assess the effectiveness of the exercise intervention. If there is a fair improvement in the lipid profile after a certain period, the exercise can be represcribed, making its effect more valuable. The lipid profile can be evaluated monthly, thereby, achieving expected results within the actual intervention period. Furthermore, this method will facilitate health practitioners in elucidating the impact of the intervention when only two markers show improvement and the other two show deterioration. Moreover, two or more interventions yielded comparable results, which allowed the selection of the most beneficial intervention. The most effective exercise intervention for optimal patient benefits was determined.

Tables 8, 9, and 10 present examples of utilising the SLIEX score from a study (11) that compared the effects of HIIT and MICT on lipid profiles following a 6-week intervention. For the HIIT intervention, the marks for changes in TG, LDL-C, HDL-C, and TC levels were 1, 1, 1, and 0, respectively. The weight of change for TG was 1 mark, LDL-C was 1 mark, HDL-C was 1 mark, and TC was 1 mark. Every weight change from baseline to post-test, from normal to normal, received one mark. The overall score for the HIIT intervention was 7 out of 18 (38.9%), indicating good improvement. Meanwhile, MICT intervention resulted in 0 marks for TG, −1 mark for LDL-C, 1 mark for HDL-C, and −1 mark for TC. A negative score indicated a worsening effect of the intervention. The weight of change for TG was 1 mark, for LDL-C was 1 mark, for HDL-C was 0 marks, and for TC was 1 mark. The total score for the MICT intervention was 2 out of 18, representing an improvement of 11.1%, which was deemed a fair improvement. This example clearly demonstrates that both interventions are effective in improving lipid profiles; however, HIIT is more effective than MICT. Consequently, health practitioners could anticipate the most beneficial interventions for patients with CAD.

Furthermore, the SLIEX tool is not only applicable for selecting the most effective interventions but can also be utilised to assess the extent to which therapy would enhance specific patients’ lipid profiles. Health practitioners can successfully convey the progress made after interventions to patients through pre- and post-evaluations. For instance, after 3 months of intervention, the score was 3 out of 18, indicating a 17% improvement. Ideally, health practitioners should engage in discussions with patients and re-evaluate prescribed interventions because fair improvement is observed. The SLIEX tool may facilitate the role of health professionals in controlling lipid levels in patients.

The SLIEX can also be used by medical professionals to assess the efficacy of prescription medications for a certain duration. For example, the SLIEX tool can be used to assess the impact of statin therapy after 3 months of administration. Thus, medical professionals can ascertain whether an anticipated goal is attainable. If the desired goal is not achieved, the prescribed dosage can be adjusted using the SLIEX tool. This may aid medical professionals in establishing the prescribed dosages of medications.

Therefore, by using SLIEX scoring, health professionals can determine which interventions significantly improve lipid profiles based on percentage of improvement. This will make it easier for health professionals to select the most appropriate interventions for patient improvement. Health professionals can use the SLIEX scoring tool to assess the effectiveness of other interventions, including individual pharmacological interventions. Hence, the SLIEX scoring tool may conclude the decision-making process for health professionals while explaining the overall benefit of the intervention in improving lipid profiles. Table 11 presents a brief description of the SLIEX scores. Clarification of each SLIEX score is provided.

### Validity and Reliability of SLIEX Score

Each observer was required to evaluate 12 articles, comprising 12 studies on HIIT and 12 on MICT, for a total of 24 interventions. The interobserver reliability of the SLIEX scoring tool ranged from substantial to almost perfect agreement, despite the observers’ varying levels of experience, and there was no provision of specific training or familiarisation for its use. This finding indicates that this scoring tool is easy to understand and straightforward for interpreting the effects of exercise interventions on lipid profiles. A follow-up discussion revealed minor oversights among the observers in providing a score for the weight of the changes. The observer agreed that the mistake occurred because of misreading the baseline category, hence, affecting the scores for weighting of changes. After consolidating the results, no further disagreements were observed. Therefore, the SLIEX score is considered an acceptable scoring tool for interpreting the effects of exercise interventions on lipid profiles.

The reliability of each observer’s total score was assessed using ICCs, and the results showed excellent reliability. Analysis of the 24 studies yielded ICCs ranging from 0.950 to 0.993. The typical difference between the observer-summated SLIEX scores ranged from 1 to 3 and was not systematically different. During the follow-up discussion, it was discovered that the error occurred due to the misjudgement of the value of the changes pre- and post-marker. Therefore, the worst-case error of the summated SLIEX score can be minimised because it is straightforward and tolerated.

To the researchers’ knowledge, no scoring tool exists to assess the overall improvement in lipid profiles after an intervention period. Thus, SLIEX is a new scoring tool that assists in interpreting the effects of exercise on four different markers of lipid profiles. Using this scoring tool, the most effective exercise intervention to improve health outcomes has been identified. Therefore, the researchers are confident that SLIEX will enhance the process of prescribing optimal exercise interventions, thereby, improving health outcomes in clinical practice.

### Potential Limitation of SLIEX Tool

This instrument evaluates the overall enhancement of lipid profiles after any intervention of a specific duration. Human mistakes are most likely to occur while adding points. To avoid such mistakes, an electronic-based computation may be required.

## Conclusion

SLIEX scoring is a new and reliable tool that assists health professionals in interpreting the effects of interventions on lipid profiles in clinical practice. The implementation of this scoring tool enables identification of the most effective intervention, which may significantly enhance health outcomes in individuals with abnormal lipid profiles. Improving lipid profiles is essential, as it may prevent the build-up of atherosclerotic plaques in epicardial coronary arteries, thereby, potentially decreasing the occurrence of adverse events in patients with CAD and other individuals with lipid abnormalities.

## Figures and Tables

**Table 1:** Effect of aerobic exercise on dyslipidaemia markers among CAD patients

No.	Author(s)	Year	Participants (*n*)	Type of medications	Exercise intervention	Dyslipidaemia risk factor			
						TG	TC	LDL-C	HDL-C
1	McGregor et al. (16)	2023	HIIT = 136 MICT= 154	Beta-blocker Anti-hypertensive Anti-platelet Statin Anti-anginal Diuretic	Frequency: 2/week (8 weeks) Intensity: HIIT: > 85% HR max MICT: 40%–70% HRR Mode: Cycle ergometer Duration: HIIT: 29–34 min MICT: 30–55 min	HIIT ↓ 4.3% MICT ↓ 9.0%	HIIT ↑ 1.7% MICT ↑ 2.0%	HIIT ↑ 0.4% MICT ↓ 0.8%	HIIT ↑ 5.7% MICT ↑ 10.1%
2	Conraads et al. (10)	2015	HIIT = 85 MICT = 89	Beta-blocker Anti-hypertensive Nitrates Diuretic Anti-arrhythmic Acetylsalicylic acid Vitamin K antagonists Digitalis Statins Antidiabetic	Frequency: 3/week (12 weeks) Intensity: HIIT: 90%–95% HR max MICT: 65%–50% HR max Mode: Bicycle Duration: HIIT: 38 min MICT: 47 min	HIIT ↓ 1.3% MICT ↓ 2.3%	HIIT ↑ 4.7% MICT ↑ 4.4%	HIIT ↑ 4.8% MICT ↑ 3.5%	HIIT ↑ 7% MICT ↑ 8.1%
3	Moholdt et al. (17)	2012	HIIT = 30 MICT = 59	β-receptor antagonist Statins ACE inhibitors/AT II-antagonist Acetylsalicylic acid	Frequency: 2/week (48 weeks) Intensity: HIIT: 85%–95% HR max MICT: vigorous Mode: treadmill Duration: HIIT: 38 min MICT: 60 min	HIIT ↑ 12.8% MICT ↓ 1.8%			HIIT ↑ 3.1% MICT ↑ 1.6%
4	Moholdt et al. (18)	2009	HIIT = 23 MICT = 25	Beta-blockers Statins Diuretic ACE inhibitors	Frequency: 5/week (24 weeks) Intensity: HIIT: 90% HR max MICT: 70% HR max Mode: treadmill Duration: HIIT: 38 min MICT: 46 min	HIIT ↓ 13.0% MICT ↓ 12.4%		HIIT ↓ 4.3% MICT ↓ 0.4%	HIIT ↑ 4.6% MICT ↑ 1.5%
5	Madssen et al. (15)	2014	HIIT = 15 MICT = 21	Aspirin Clopidogrel Statins Beta-blockers ACE inhibitors/AT II-antagonist	Frequency: 3/week (12 weeks) Intensity: HIIT: 85%–95% HR max MICT: 70% HR max Mode: treadmill Duration: HIIT: 38 min MICT: 46 min	HIIT ↑ 9.0% MICT ↑ 8.0%	HIIT ↓ 2.3% No changes after MICT	HIIT ↓ 8.3% MICT ↓ 4.2%	No changes after HIIT MICT ↑ 7.7%
6	Kim et al. (13)	2015	HIIT = 14 MICT = 14	Acetylsalicylic acid Statins Beta-blockers ACE inhibitors/AT II-antagonist	Frequency: 3/week (6 weeks) Intensity: HIIT: 85%–95% HRR MICT: 70%–85% HRR Mode: treadmill Duration: HIIT: 45 min MICT: 45 min	HIIT ↑ 4.0% MICT ↑ 17.8%		HIIT ↓ 37.7% MICT ↓ 27.1%	No changes after HIIT and MICT
7	Dunford et al. (11)	2021	HIIT = 9 MICT = 9	Beta-blockers ACE inhibitors Acetylsalicylic acid Lipid lowering Metformin	Frequency: 3/week (6 weeks) Intensity: HIIT: 85%–95% HRR MICT: 70%–85% HRR Mode: stair climbing vs. treadmill, cycling and walking Duration: HIIT: 45 min MICT: 45 min	HIIT ↓ 6.0% No changes after MICT	No changes after HIIT MICT ↑ 11.1%	HIIT ↓ 7.1% MICT ↑ 23.0%	HIIT ↑ 8.3% MICT ↑ 16.3%
8	Abdelhalem et al. (9)	2018	HIIT = 20 MICT = 20	NR	Frequency: 2/week (12 weeks) Intensity: HIIT: 85%–95% HRR MICT: 40%–60% HRR Mode: treadmill Duration: HIIT: 40–45 min MICT: 40–45 min	HIIT ↓ 14.1% MICT ↓ 13.4%	HIIT ↓ 11.8% MICT ↓ 10.1%	HIIT ↓ 10.6% MICT ↓ 12.8%	HIIT ↑ 17.7% MICT ↓ 6.7%
9	Taylor et al. (20)	2022	HIIT = 34 MICT = 39	Calcium channel blockers Statins Beta-blockers ACE inhibitors Diuretic Antiarrhythmic Anticoagulant Aspirin Other antiplatelet	Frequency: 3/week (32 weeks) Intensity: HIIT: 15–18 RPE MICT: 11–13 RPE Mode: treadmill and bike Duration: HIIT: 33–35 min MICT: 40 min	HIIT ↓ 7.8% MICT ↑ 8.4%	No changes after HIIT MICT ↑ 8.2%	HIIT ↓ 5.5% MICT ↓ 5.3%	HIIT ↑ 7.7% MICT ↑ 7.7%
10	Lee et al. (14)	2019	HIIT = 7 MICT = 7	Statins Beta-blockers ACE inhibitors	Frequency: 3/week (24 weeks) Intensity: HIIT: 90%–95% HR max MICT: 60%–80% VO_2_ max Mode: walking and jogging Duration: HIIT: 35–45 min MICT: 60 min	No changes after HIIT MICT ↓ 6.2%		HIIT ↓ 7.7% No changes after MICT	HIIT ↓ 7.1% No changes after MICT
11	Gonçalves et al. (12)	2024	HIIT = 23 MICT = 23	ACE inhibitor AT II-antagonist Antiplatelet Calcium channel blockers Beta-blockers Diuretic Insulin Statin	Frequency: 3/week (6 weeks) Intensity: HIIT: 85%–95% HR max MICT: 70%–75% HR max Mode: treadmill Duration: HIIT: 34 min MICT: 43 min	HIIT ↓ 31.4% MICT ↓ 26.5%	HIIT ↓ 14.1% MICT ↓ 13.3%	HIIT ↓ 27.4% MICT ↓ 23.5%	HIIT ↑ 26.1% MICT ↑ 20.7%
12	Pernick (19)	2017	HIIT = 31 MICT = 30	NR	Frequency: 4/week (32 weeks) Intensity: HIIT: 60%–85% VO_2_ max MICT: 50%–60% VO_2_ max Mode: NR Duration: HIIT: 50 min MICT: 41 min	HIIT ↓ 0.9% MICT ↑ 5.1%	HIIT ↑ 5.0% MICT ↑ 6.6%	HIIT ↓ 6.3% MICT ↑ 8.3%	HIIT ↑ 9.0% MICT ↑ 5.4%

Notes: HR = heart rate; HRR = heart rate reserve; ACE = angiotensin-converting enzyme inhibitors; AT II = Angiotensin II Receptor Antagonist; HIIT = high intensity interval training; MICT = moderate intensity continuous training; NR = not reported; RPE = rating perceived exertion; VO_2_ = volume oxygen; ↑ = increase; ↓ = decrease

**Table 2:** Norm for dyslipidaemia markers

Category	TG	LDL-C	HDL-C	TC
Normal	< 1.69 mmol/L	< 2.58 mmol/L	≥ 1.05 mmol/L	< 5.17 mmol/L
	< 150 mg.dL	< 100 mg.dL	≥ 41 mg.dL	< 200 mg.dL

Near optimal/above optimal	Not related	2.58–3.34 mmol/L	Not related	Not related
		100–129 mg.dL		

Borderline high	1.69–2.24 mmol/L	3.35–4.12 mmol/L	≤ 1.04 mmol/L	5.17–6.19 mmol/L
	150–199 mg.dL	130–159 mg.dL	≤ 40 mg.dL	200–239 mg.dL

High	2.25–5.63 mmol/L	4.13–4.90 mmol/L	Not related	6.20 mmol/L
	200–499 mg.dL	160–189 mg.dL		≥ 240 mg.dL

Very high	≥ 5.64 mmol/L	4.91 mmol/L	Not related	Not related
	≥ 500 mg.dL	≥ 190 mg.dL		

Note: Adopted from the Third Report of the National Cholesterol Education Program (NCEP) Expert Panel on Detection, Evaluation, and Treatment of High Blood Cholesterol in Adults (Adult Treatment Panel III) (23)

**Table 3:** Scoring for weightage changes for TG

	Normal	Borderline high	High	Very high
Normal	+1	−1	−2	−3
Borderline high	+2	+1	−1	−2
High	+3	+2	+1	−1
Very high	+4	+3	+2	+1

**Table 4:** Scoring for weightage changes for LDL-C

	Normal	Near optimal	Borderline high	High	Very high
Normal	+1	−1	−2	−3	−4
Near optimal	+2	+1	−1	−2	−3
Borderline high	+3	+2	+1	−1	−2
High	+4	+3	+2	+1	−1
Very high	+5	+4	+3	+2	+1

**Table 5:** Scoring for weightage changes for HDL-C

	Normal	Abnormal
Normal	+1	−1
Abnormal	+2	0

**Table 6:** Scoring for weightage changes for TC

	Normal	Borderline high	High
Normal	+1	−1	−2
Borderline high	+2	+1	−1
High	+3	+2	+1

**Table 7:** Inter-observer reliability (Kappa ± standard error) between three expert reviewers

SLEIX criteria		Observer 1 vs. observer 2	Observer 2 vs. observer 3	Observer 1 vs. observer 3
Changes pre and post	TG	1.000 (0.000)^a^	1.000 (0.000)^a^	1.000 (0.000)^a^
	TC	1.000 (0.000)^a^	1.000 (0.000)^a^	1.000 (0.000)^a^
	LDL-C	1.000 (0.000)^a^	1.000 (0.000)^a^	1.000 (0.000)^a^
	HDL-C	0.793 (0.138)^b^	1.000 (0.000)^a^	0.793 (0.138)^b^

Weightage of changes	TG	0.733 (0.185)^b^	0.733 (0.185)^b^	1.000 (0.000)^a^
	TC	Constant	Constant	Constant
	LDL-C	1.000 (0.000)^a^	1.000 (0.000)^a^	1.000 (0.000)^a^
	HDL-C	0.899 (0.091)^a^	0.899 (0.091)^a^	1.000 (0.000)^a^

Notes: Superscript letters denote the following level of agreement between observers: a = almost perfect; b = substantial; constant = 100% agreement

**Table 8:** Example of results of HIIT and MICT on lipid profiles

Intervention	Lipid profile (mmol/L)	Pre (mmol/L)	Post (mmol/L)
HIIT	TG	0.86	0.80
	LDL-C	1.40	1.30
	HDL-C	1.20	1.30
	TC	3.00	3.00

MICT	TG	1.10	1.10
	LDL-C	1.30	1.60
	HDL-C	0.86	1.00
	TC	2.70	3.00

**Table 9:** Scoring sheet for effect of HIIT and MICT on lipid profiles

Intervention	Changes pre- and post-test				∑	Weightage changes (refer table weightage)				∑	Total score	% of improvement
	
	TG	LDL	HDL	TC		TG	LDL	HDL	TC			
HIIT	+1	+1	+1	0	3	+1	+1	+1	+1	4	7	38.9
MICT	0	−1	+1	−1	−1	+1	+1	0	+1	3	2	11.1

**Table 10:** Classification of improvement of lipid profiles

Percentage of improvement	Classification
< 25	Fair
25–50	Good
> 50–75	Very good
> 75	Excellent

**Table 11:** Detailed SLIEX score

Criteria	Explanation	Scoring
**Changes pre and post**		
Changes of TG after intervention	Positive changes when the TG values improved/decrease following the completion of the intervention. There are no changes when the pre-test and post-test results are identical. Negative changes when the TG values worsen/increase following the completion of the intervention.	TG decrease; score +1 positive change TG remains; score 0 minimal benefit TG increase; score −1 negative change
Changes pre and post-LDL-C	Positive changes when the LDL-C values improved/decrease following the completion of the intervention. There are no changes when the pre-test and post-test results are identical. Negative changes when the LDL-C values worsen/increase following the completion of the intervention.	LDL-C decrease; score +1 positive change LDL-C remains; score 0 minimal benefit LDL-C increase; score −1 negative change
Changes pre and post-HDL-C	Positive changes when the HDL-C values improved/decrease following the completion of the intervention. There are no changes when the pre-test and post-test results are identical. Negative changes when the HDL-C values worsen/increase following the completion of the intervention.	HDL-C decrease; score +1 positive change HDL-C remains; score 0 minimal benefit HDL-C increase; score −1 negative change
Changes pre and post-TC	Positive changes when the TC values improved/decrease following the completion of the intervention. There are no changes when the pre-test and post-test results are identical. Negative changes when the TC values worsen/increase following the completion of the intervention.	TC decrease; score +1 positive change TC remains; score 0 minimal benefit TC increase; score −1 negative change
		**Total score for changes = −4 to +4**
**Weightage changes**		
Weightage changes TG	+1 score is given when no changes category of TG from pre-test to post-test. +2 score is given when improved in 1 category from pre-test to post-test. +3 score is given when improved in 2 categories from pre-test to post-test. +4 score is given when improved in 3 categories from pre-test to post-test. Refer Table 3. The total score of TG was −3 to +4.	No changes in category; score +1 1 category improvement; score +2 2 categories improvement; score +3 3 categories improvement; score +4 1 category worsen; score −1 2 categories worsen; score −2 3 categories worsen; score −3
Weightage changes LDL-C	+1 score is given when no changes category of LDL-C from pre-test to post-test. +2 score is given when improved in 1 category from pre-test to post-test. +3 score is given when improved in 2 categories from pre-test to post-test. +4 score is given when improved in 3 categories from pre-test to post-test. +5 score is given when improved in 4 categories from pre-test to post-test. Refer Table 4. The total score of LDL-C was −4 to +5.	No changes in category; score +1 1 category improvement; score +2 2 categories improvement; score +3 3 categories improvement; score +4 3 categories improvement; score +5 1 category worsen; score −1 2 categories worsen; score −2 3 categories worsen; score −3 4 categories worsen; score −4
Weightage changes HDL-C	+1 score is given when no changes category of HDL-C from pre-test to post-test. +2 score is given when improved in 1 category from pre-test to post-test. Refer Table 5. The total score of HDL-C was −1 to +2.	No changes in category; score +1 1 category improvement; score +2 1 category worsen; score −1 Remain worsen; score 0
Weightage changes TC	+1 score is given when no changes category of TC from pre-test to post-test. +2 score is given when improved in 1 category from pre-test to post-test. +3 score is given when improved in 2 categories from pre-test to post test. Refer Table 6. The total score of TC was −2 to +3.	No changes in category; score +1 1 category improvement; score +2 2 categories improvement; score +3 1 category worsen; score −1 2 categories worsen; score −2
		**Total score for weightage changes = −4 to +14**
		**Total SLIEX score = 18 points**
